# A Comparative Metabolomic Analysis Reveals the Nutritional and Therapeutic Potential of Grains of the Traditional Rice Variety Mappillai Samba

**DOI:** 10.3390/plants11040543

**Published:** 2022-02-18

**Authors:** Veera Ranjani Rajagopalan, Sudha Manickam, Raveendran Muthurajan

**Affiliations:** Department of Plant Biotechnology, Tamil Nadu Agricultural University, Coimbatore 641003, Tamil Nadu, India; rajaranji@gmail.com (V.R.R.); sudhatamil@gmail.com (S.M.)

**Keywords:** traditional rice, Mappillai Samba, metabolomics, therapeutic compounds, phytosterols, antioxidants

## Abstract

Rice (*Oryza sativa* L.) is the staple food of the majority of the population, particularly in Asia and Africa. Enriching rice with nutritional and therapeutic contents can improve its benefits for patients with lifestyle disorders. This study aimed to profile the phytochemical contents of the therapeutically known traditional rice Mappillai Samba against white rice CBMAS 14065 using non-targeted gas chromatography–mass spectrometry (GC-MS/MS). An analysis of the data using a mass spectrometry–data independent analysis (MS-DIAL) and MetaboAnalyst identified 113 metabolites belonging to 21 different classes of metabolites. A partial least square-discriminant analysis (PLS-DA) revealed 43 variable importance in projection (VIP) metabolites. This study identified therapeutically important metabolites, including phenylpropanoids, phytosterols, flavonoids, and polyamines, in the grains of Mappillai Samba. Three significant metabolic pathways, viz., phenylpropanoid biosynthesis, ubiquinone and other terpenoid-quinone biosynthesis, and steroid biosynthesis, were responsible for the grain metabolome variation between CBMAS 14065 and Mappillai Samba. Overall, the results of this study unravelled the biochemical complexity of Mappillai Samba, paving the way for the genetic mapping of the therapeutic compound accumulation in rice and the development of similar therapeutic rice varieties through molecular breeding.

## 1. Introduction

Rice is one of the most important staple foods of nearly more than half of the global population, and the majority of Asians consume rice thrice a day. Rice production has increased by >117% (from 257 million tons in the 1960s to 570 million tons in 2000) through the introduction of high yielding semi-dwarf varieties and hybrids [1]. Although rice is considered to be a high energy and high calorie cereal, its grains contain low amounts of protein, minerals, and nutrients [2]. Increased rice consumption is related to the prevalence of diabetes, which in turn leads to a susceptibility to neurological complications, cardiovascular diseases, retinopathy, foot ulcers, and renal diseases [3]. It has also been reported to aggravate the prevalence of anaemia in women. After the first green revolution, rice improvement programs were mainly aimed at increasing yields, with minimal attention paid toward improving the nutritional quality. The global population is predicted to reach 9.7 billion by 2050 [4], and achieving food and nutritional security for the growing population is becoming a challenge. Considering its widespread consumption, the development and dissemination of rice with nutritional and therapeutic values may have a significant impact on the general health and nutritional status of rural women and children where rice is the major source of daily calorie needs.

High yielding rice varieties do not possess high contents of the nutritional and therapeutic phytochemicals that are abundant in traditional rice varieties and landraces. Several traditional rice genotypes have been reported to possess nutritional/therapeutic phytochemicals such as flavonoids, anthocyanins, γ-oryzanol, phytosterols, GABA, tocopherol, tricin, and carotenoids (lutein and beta-carotene), which attenuate the incidence of non-communicable diseases (cardiovascular diseases, diabetes, retinopathy, cancer, and strokes) and help in maintaining the general health of pregnant women and children [5,6,7,8]. Traditional rice genotypes, viz., Njavara from Kerala [9,10] and Kavuni from Tamil Nadu [11], are known for their antidiabetic, anticancer, and other medicinal/therapeutic properties. The consumption of biofortified rice varieties with the nutritional and therapeutic values of traditional rice may help in managing health issues such as obesity, hypertension, diabetes, and oedemas [12,13].

The development of biofortified rice varieties requires a thorough understanding of genetic variation in the accumulation of metabolites. Non-targeted metabolomics allows us to profile the majority of secondary metabolites in plants. An assessment of nutritional and bioactive phytochemicals through targeted LC-MS/MS in selected scented and pigmented rice varieties has revealed the presence of 4-hydroxy benzoic acid, apigenin, tricin, avenasterol, coumarin, coumaric acid, phenyl alanine, caffeic acid, and α-tocophenol [14]. In this study, a non-targeted metabolomics approach was used to analyse the metabolomic profiles of a popular traditional rice variety, Mappillai Samba (Bridegroom’s Rice) and a high yielding white rice genotype, type CBMAS 14065. Mappillai Samba (also known as Bridegroom’s Rice), a red rice variety of Tamil Nadu, India, is known for its health benefits. Independent studies have reported its antihypercholesterolemic effect, anticancer activity, ability to improve fertility in men [15] as well as antidiabetic [16] and antineurological properties [17]. However, no systematic studies have been conducted to unravel the metabolome complexity of Mappillai Samba, which would allow us to map these traits and use them for breeding applications.

## 2. Results

### 2.1. Characteristics of Mappillai Samba and CBMAS 14065

**Figure 1:**
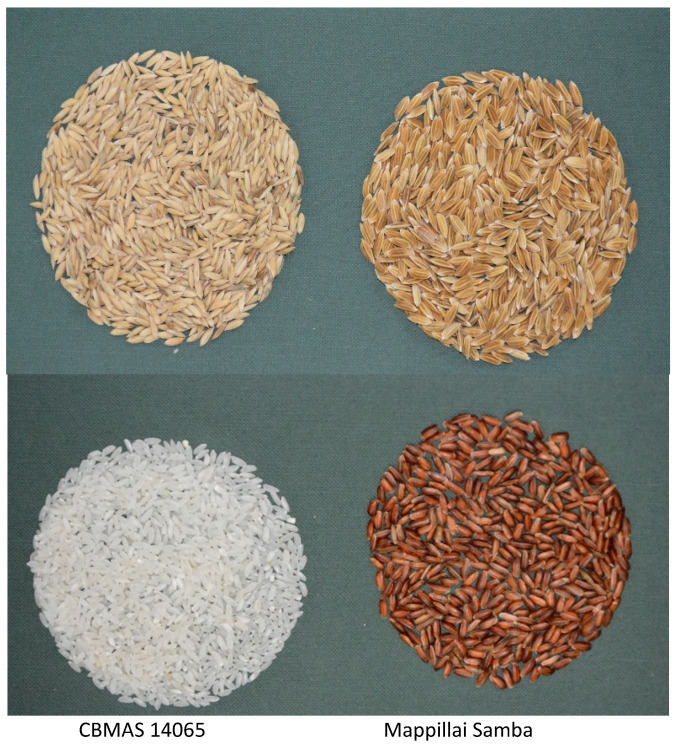
Grain characteristics of the rice genotypes CBMAS 14065 and Mappillai Samba.

Mappillai Samba is a long-duration rice genotype with bold, red grains whereas CBMAS 14065 is a medium-duration rice variety with white, medium-slender grains (Table 1; Figure 1).

### 2.2. Metabolite Profiles of the Contrasting Rice Genotypes

**Figure 2:**
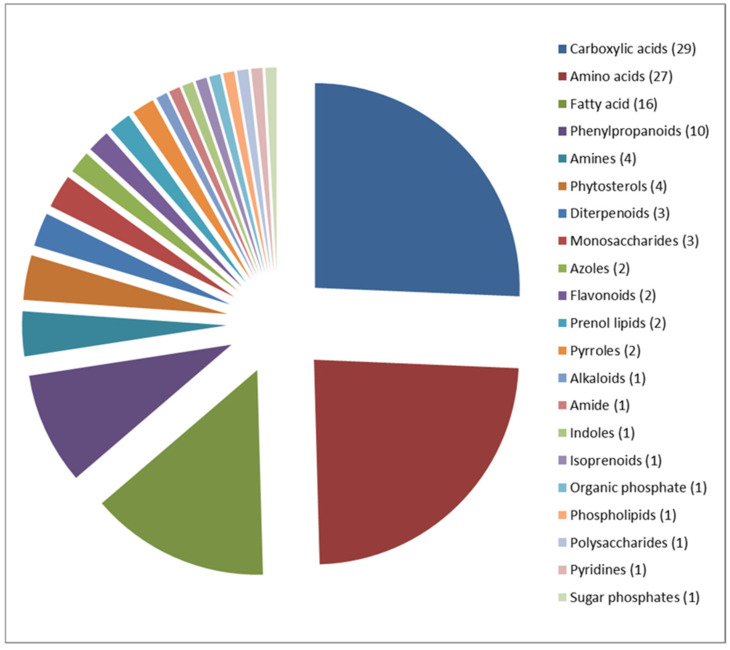
Classification of 113 metabolites identified in the grains of Mappillai Samba and CBMAS 14065 into 21 different metabolite classes. The number in parentheses indicates the number of metabolites mapped against each metabolite class.

**Figure 3:**
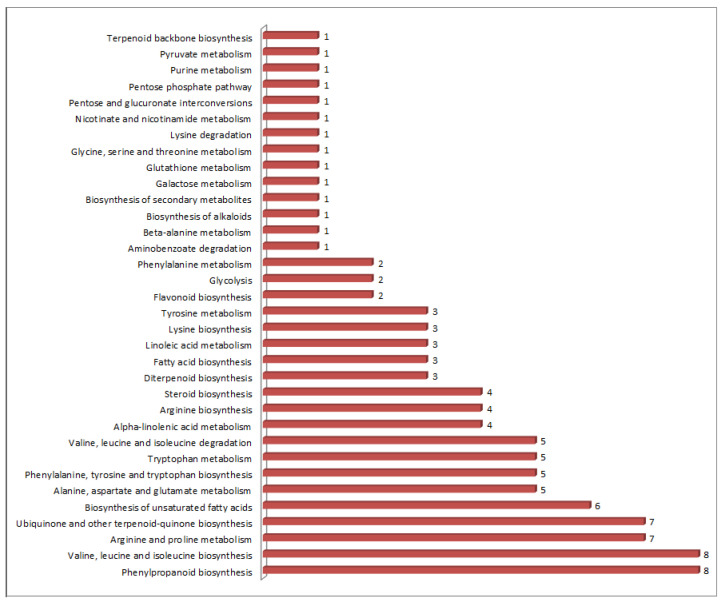
Mapping of 113 different metabolites belonging to 21 different classes against 34 sub-pathways listed in KEGG (Kyoto Encyclopaedia of Genes and Genomes). Values indicate the number of metabolites mapped against the respective pathway.

The metabolomic profiling of the grains of Mappillai Samba and CBMAS 14065 identified 113 known compounds (Supplementary Table S1 and Figure S1) belonging to 21 different metabolite classes, viz., carboxylic acids (29 compounds), amino acids (27 compounds), fatty acids (16 compounds), and phenylpropanoids (10 compounds) (Figure 2). The mapping of these metabolites against 34 different sub-pathways listed in KEGG revealed that the majority of these metabolites are involved in phenylpropanoid biosynthesis (8); valine, leucine, and isoleucine biosynthesis (8); arginine and proline biosynthesis (7); and ubiquinone and other terpenoid-quinone biosynthesis (7) (Figure 3).

### 2.3. Univariate and Multivariate Analyses

**Figure 4:**
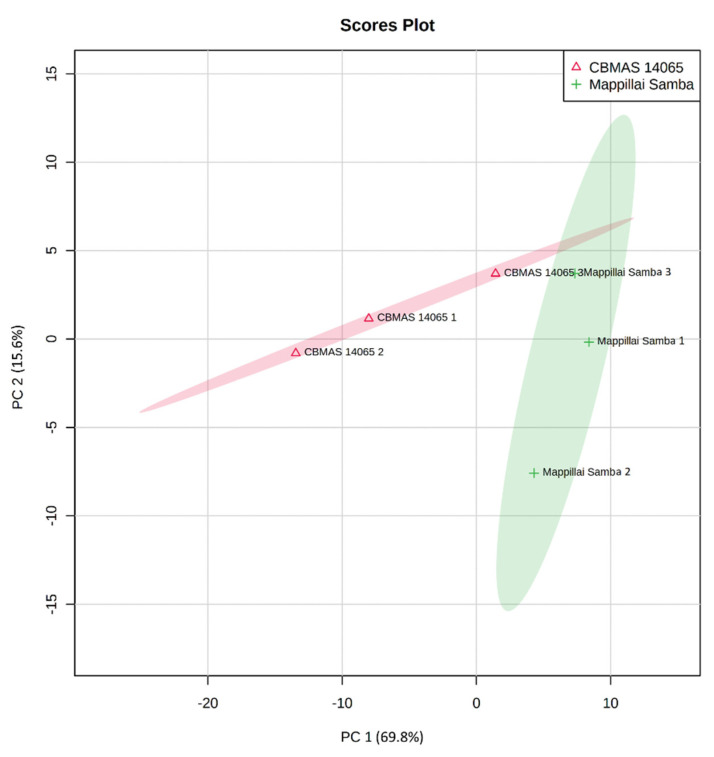
Principal component analysis showing the uniqueness of grain metabolomic profiles between Mappillai Samba and CBMAS 14065. PC1 explained a variation of 69.8% and PC2 explained a 15.6% variation (cumulative variance of 85.4%).

**Figure 5:**
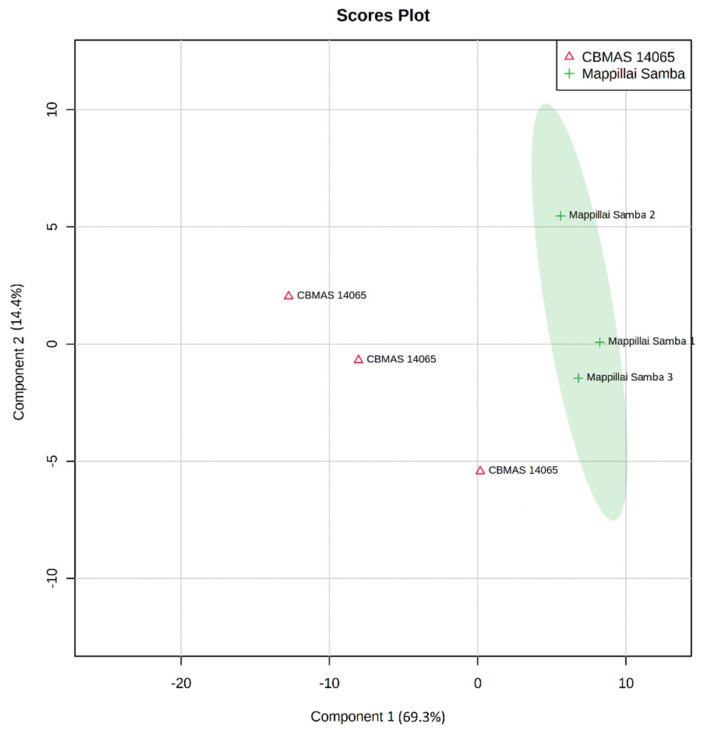
Partial least-square discriminant analysis discriminating Mappillai Samba and CBMAS 14065 based on the grain metabolome (cumulative variance of 83.7%; 69.3% and 14.4% variance explained by components 1 and 2, respectively).

Hierarchical clustering based on the metabolite profiles of Mappillai Samba and CBMAS 14065 revealed distinct differences between the two genotypes. A principal component analysis (PCA) provided an initial estimate of the metabolic differences between the red grain Mappillai Samba and the white grain CBMAS 14065. The results of the PCA revealed that the first component PC1 explained 69.8% of the variance and PC2 explained 15.6% of the cumulative variance of 85.4% (Figure 4). The results of the partial least square-discriminant analysis (PLS-DA) also captured similar differences with a cumulative variance of 83.7% (component 1 explaining 69.3% and component 2 explaining 14.4% of the variance) (Figure 5).

**Figure 6:**
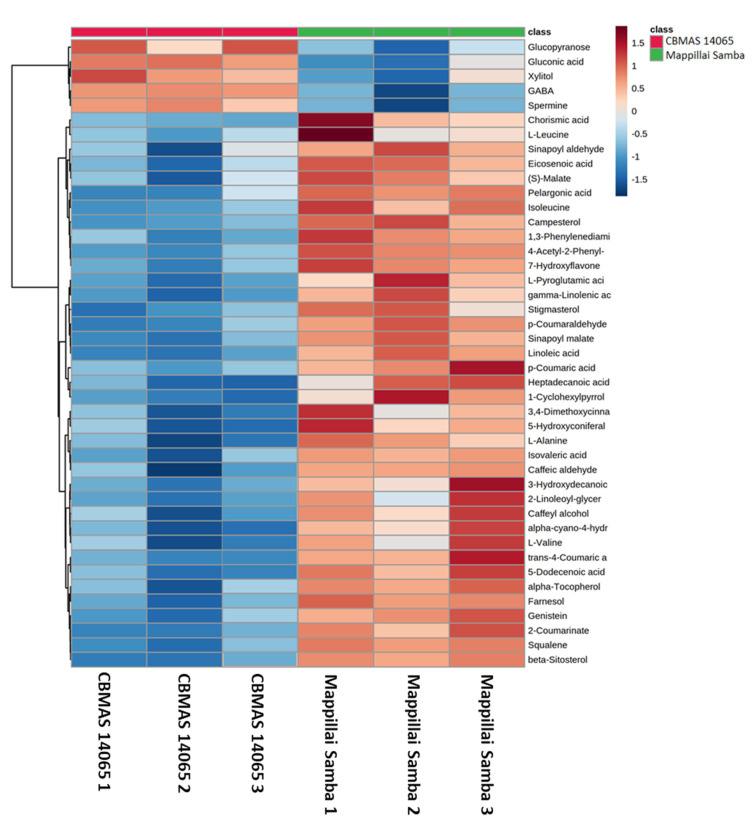
Heat map analysis showing the abundance of PLS-DA VIP metabolites between the grains of Mappillai Samba and CBMAS 14065.

Among the 113 metabolites annotated, the PLS-DA identified 43 VIP metabolites discriminating the grains of Mappillai Samba and CBMAS 14065 with a VIP score of > 1 (Table 2; Figure 6). A heat map of the VIP metabolites revealed that the therapeutic metabolites phenylpropanoids, flavonoids, phytosterols, and amino acids were abundant in the grains of Mappillai Samba whereas stress-related amino acids and sugar compounds, including spermine, GABA, xylitol, gluconic acid, and glucopyranose, were abundant in the grains of CBMAS 14065 (Figure 6). A heat map analysis of all the identified 113 metabolites (Supplementary Figure S2) revealed that therapeutic metabolites were abundant in the grains of Mappillai Samba whereas stress-related amino acids and sugars were abundant in the grains of the white rice cultivar CBMAS 14065.

### 2.4. Fold Change Analysis

An estimation of the abundance ratio of 113 metabolites between the grains of Mappillai Samba and CBMAS 14065 revealed that 84 metabolites were upregulated and 8 metabolites were downregulated in the grains of Mappillai Samba (Supplementary Table S2). Most of the downregulated metabolites in the grains of Mappillai Samba were stress-related amino acids and sugars. The majority of the upregulated metabolites in the grains of Mappillai Samba were therapeutic metabolites, including phenylpropanoids, phytosterols, flavonoids, and amino acids.

### 2.5. Pathway Analysis

**Figure 7:**
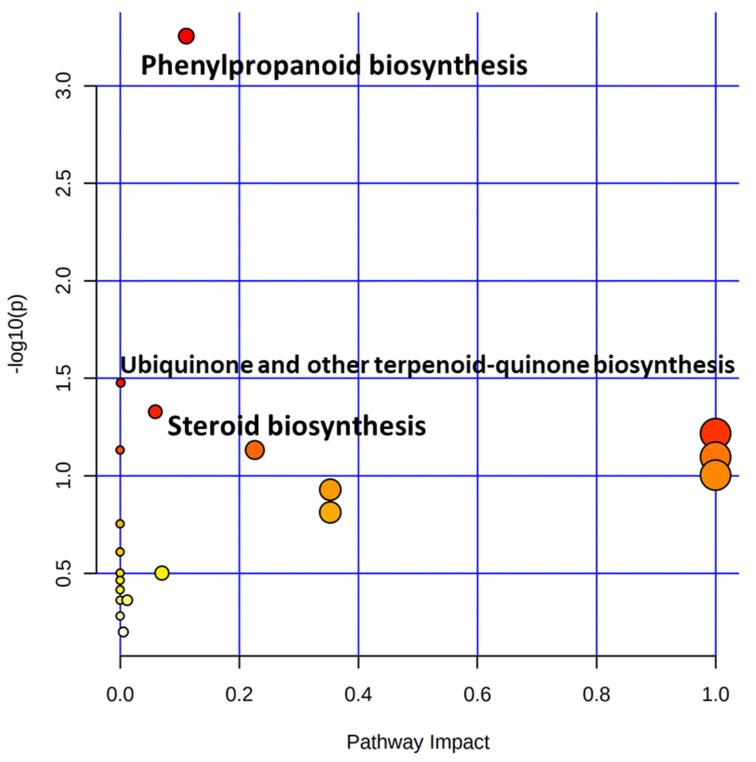
Metabolic pathways differentiating the grains of Mappillai Samba and CBMAS 14065. Each circle represents a metabolic pathway; the red circle indicates a higher impact and the yellow one indicates a lower impact. The size of the circle represents the number of differential metabolites present in the pathway.

The pathway mapping of significant metabolites against the KEGG database identified three significant metabolic pathways with FDR values ≤0.05. The phenylpropanoid pathway showed the highest −log (*p*) value of 3.2552, followed by the ubiquinone and other terpenoid-quinone biosynthesis pathway (1.4771) and the steroid biosynthesis pathway (1.3282) (Table 3 and Figure 7).

## 3. Discussion

Safeguarding the overall health status of humans requires an adequate intake of a nutritious and balanced diet. Fortifying a regular staple food crop such as rice with essential nutrients through genetic improvements can provide a sustainable solution. The development of rice with nutritional and therapeutic compounds could have a significant impact on the general health and nutritional status of the rural populations in India where rice remains the major source of daily calorie needs. The recently developed high yielding rice varieties do not possess the high nutritional content and therapeutic phytochemicals that are abundant in traditional rice varieties. A traditional rice genotype, Njavara of Kerala, has been reported to possess antidiabetic and anticancerous properties [9,10]. Similarly, a traditional rice genotype, Kavuni, was reported to possess therapeutic compounds that circumvented diabetes and age-related macular degeneration [11]. Similar other attempts have been made and rice varieties possessing anti-inflammatory, anticancer, and antioxidant properties have been reported [15,18].

Advanced molecular methods have been developed to analyse the transcriptomes, proteomes, and metabolomes of crop plants [19]. Plant metabolomics through the targeted and non-targeted profiling of the metabolome has permitted a quality analysis [20,21], drug discovery [22], the detection of GM products [23], and the detection of biohazards [24]. A large-scale metabolomic study using GC-MS and UHPLC-MS/MS in a set of diverse rice genotypes (comprising both *japonica* and *indica* cultivars) identified 121 metabolites that significantly differentiated the two subspecies. Metabolomic data clearly indicated that the grains of *indica* rice contained increased levels of γ-tocopherol, γ-tocotrienol, and pyridoxate but reduced levels of phytic acid, gluconate, and nicotianamine compared with the grains of *japonica* [25]. In another study, a GC-MS/UHPLC-MS analysis of rice grains led to the identification of 214 metabolites [26]. A metabolomic analysis of the giant embryo rice Shangshida No. 5 revealed the presence of high levels of bioactive compounds such as GABA, arabinose, β-alanine, glutathione, VB6, xylitol, and xylose [27]. It was also demonstrated that *indica* and *japonica* rice considerably differed in their grain flavonoid composition, which was attributed to the sequence variations in the genes involved in flavonoid biosynthesis [28].

The present study aimed to unravel the grain metabolome complexity of the traditional rice variety Mappillai Samba, which is known for its nutritional and therapeutic properties. Mappillai means newlywed bridegroom and this rice (Mappillai Samba) is named after the reason that it is capable of improving the health and body power of Mappillai. A few studies have attempted to target the quantification of carbohydrates, proteins, and antioxidants in the grains of Mappillai Samba but there is no holistic study analysing its secondary metabolite composition [15,16,17]. In this study, the grain metabolites of Mappillai Samba and the white rice genotype CBMAS 14065 were profiled using GC-MS/MS.

A GC-MS/MS analysis of the grains of Mappillai Samba and CBMAS 14065 revealed a total of 113 metabolites (Supplementary Table S1) belonging to 21 different metabolite classes mapped onto 34 different KEGG pathways (Figure 3). Of these 113 metabolites, 43 were found to differentiate the grains of Mappillai Samba and CBMAS 14065 (Figure 6). The PCA results revealed a distinct separation of the two rice varieties based on their metabolite contents. A fold change analysis revealed that therapeutic metabolites such as phenylpropanoids, phytosterols, flavonoids, and amino acids were upregulated whereas stress-related amino acids and sugars were downregulated in the grains of Mappillai Samba, as reported earlier [15,16,18].

The PLS-DA identified a total of 43 significant metabolites (VIP score > 1) belonging to phenylpropanoids and phytosterols, which are known for their antihypercholesterolemic, anti-infertility, antioxidant, and anticancer properties [15,18]. Heat map and fold change analyses also confirmed the abundance of phenylpropanoids, steroids, and a few other therapeutic metabolites in the grains of Mappillai Samba compared with those in the grains of CBMAS 14065. Phytosterols such as β-sitosterol, stigmasterol, and campesterol—which are naturally found in vegetable oils, nuts, legumes, whole grains, and fruit—have cholesterol-lowering, antioxidant, and anticancer properties [29]. In this study, all three important phytosterols, viz., campesterol (92-fold increase), stigmasterol (33-fold increase), and β-sitosterol (8.5-fold increase) were found to be more abundant in the grains of Mappillai Samba than in those of CBMAS 14065. Campesterol (with antioxidant and hypocholesterolemic effects) [18], β-sitosterol (with hypocholesterolemic, antisterility, and anticancerous effects) [30], and stigmasterol (used as a precursor in the production of semi-synthetic progesterone [31] and vitamin D3) [32] are known for their valuable health-benefiting properties. Stigmasterol has also been reported to exhibit antihepatotoxic, anti-inflammatory, antioxidant, antiviral, anticancer, and antihypercholesterolemic effects [15,18]. Another pharmaceutically important terpenoid compound, squalene—which possesses antioxidant, anticancer, immunostimulant, and lipoxygenase inhibitor activities—was also found to be abundant (29-fold) in the grains of Mappillai Samba [33]. Furthermore, Mappillai Samba grains were found to contain elevated levels of gamma-tocotrienol (133-fold) and alpha-tocopherol (76-fold). To the best of our knowledge, this is the first time that gamma-tocotrienol and alpha-tocopherol have been reported in the grains of Mappillai Samba. These significant therapeutic compounds in the grains of Mappillai Samba (Table 4) may be targeted for genetic mapping to facilitate the introgression of these traits into other popular varieties. In plants, polyamines have been reported to play important roles in fundamental cellular processes such as growth, differentiation, morphogenesis, and host defence [34]. In the current study, major polyamines such as putrescine and spermine were detected in the grains of Mappillai Samba [35]. The pathway analysis predicted that the grain metabolomic variation between the two genotypes was greatly contributed to by the phenylpropanoid pathway (−log (*p*) = 3.2552), followed by the pathways for ubiquinone and terpenoid-quinone biosynthesis (−log (*p*) = 1.4771) and steroid biosynthesis (−log (*p*) = 1.3282). Overall, the present study sheds light on metabolome complexity in the grains of the traditional rice, Mappillai Samba.

## 4. Materials and Methods

### 4.1. Seed Material

A traditional red rice genotype (Mappillai Samba) and a high yielding white rice genotype (CBMAS 14065) were used. Both genotypes were raised during Rabi (2020) at the Department of Rice, Tamil Nadu Agricultural University, Coimbatore, India. Freshly harvested rice seeds were dried, manually dehusked, and used for the GC-MS/MS analysis.

### 4.2. Metabolite Extraction and Mass Spectrometric Analysis

The secondary metabolites were extracted from the grains of CBMAS 14065 and Mappillai Samba using the Soxhlet extraction procedure as described previously [47]. The dehusked seeds of Mappillai Samba and CBMAS 14065 were pulverised using liquid nitrogen in a mortar and pestle. Approximately 25 mg of the powdered rice sample was soaked in 100% methanol (HPLC grade) overnight. The mixture was kept in a water bath at 70 °C for 10 min, followed by centrifugation at 13,000× *g* for 10 min at 4 °C. The supernatant was collected and filtered through a 0.2 micron filter. The filtered extracts were concentrated using a vacuum evaporator and vials containing 1 mL of the concentrated filtrate were fed into the mass spectrometer. A GC-MS/MS (Perkin Elmer Inc., Akron, OH, USA) instrument coupled with a DB-5 MS capillary standard non-polar column (30 Mts, ID: 0.25 mm, film: 0.25 IM, (Perkin Elmer Inc, Akron, OH, USA)), available at the Department of Agricultural Microbiology, Tamil Nadu Agricultural University, Coimbatore, India, was used. One microlitre of the methanolic extract of the sample was injected into the GC-MS/MS system with helium as the carrier gas. The peaks were detected for 30 min. Toward the end of each run, an extremely high temperature (260 °C) was maintained for approximately 5 min, followed by washing the syringe (thrice) with the solvent (methanol) and equilibration (2–3 min) to ensure the avoidance of contamination. The GC-MS/MS analysis was performed with a mass range scan of 50–1000 m/z; 70 eV was applied for the fragmentation and the precursor ions were isolated with an isolation window of 10 m/z. The obtained raw mass spectra were converted to an ABF format using an ABF converter (www.reifycs.com/AbfConverter, 25 November 2021) for a further analysis.

### 4.3. Statistical Analysis and Pathway Mapping

The spectral peak processing and annotation were performed using MS-DIAL [48] as described previously [47]. The PCA and PLS-DA analyses were performed using MetaboAnalyst 5.0 (https://www.metaboanalyst.ca/, 29 November 2021) [49] with the missing values being replaced by 1/5 of the minimum positive values. The significant metabolites that discriminated Mappillai Samba and CBMAS 14065 were mapped onto metabolic pathways using MetaboAnalyst 5.0 [50]. The significant pathways that differed between Mappillai Samba and CBMAS 14065 were identified using the Kyoto Encyclopedia of Genes and Genomes (KEGG) database [51].

## 5. Conclusions

The present study aimed to generate the metabolic signatures of the traditional rice Mappillai Samba, which is known for its therapeutic value and health benefits. A GC-MS/MS analysis identified 113 metabolites belonging to 21 different metabolite classes. PCA and PLS-DA analyses identified 43 unique metabolites that differentiated the grains of the two cultivars Mappillai Samba and CBMAS 14065. Pathway mapping revealed three significant pathways, viz., the phenylpropanoid biosynthesis pathway, the pathway for ubiquinone and other terpenoid-quinone biosynthesis, and the steroid biosynthesis pathway, contributing to the grain metabolome variation between Mappillai Samba and CBMAS 14065. A fold change analysis revealed that metabolites of nutraceutical and therapeutic importance—namely, phytosterols, squalene, and tocopherol—were more abundant in the grains of Mappillai Samba than in those of CBMAS 14065. Improving the traditional rice genotypes for yield and agronomic traits or improving the high yielding varieties for phytochemical contents may be potential strategies for the biofortification of staple food crops such as rice to circumvent major lifestyle disorders. The identified therapeutic/nutraceutical compounds in the grains of Mappillai Samba may serve as potential leads for genetic mapping of these pharmaceutically important traits, which will accelerate the development of high yielding rice varieties enriched with nutritional/therapeutic traits.

## Figures and Tables

**Table 1:** Details of the agronomic characteristics of rice genotypes CBMAS 14065 and Mappillai Samba.

Cultivar	Origin	Days to Maturity	Pedigree	Features
Mappillai Samba	Traditional variety, Tamil Nadu, India	155 to 160 days	Unknown	Brownish kernel
CBMAS 14065 (Pre-release cultivar)	TNAU, Coimbatore, India	130 to 135 days	White Ponni x Apo	Drought tolerant, high yielding, fine quality, white kernel

**Table 2:** List of the significant metabolites differentiating the grains of Mappillai Samba and CBMAS 14065 based on the VIP scores of the PLS-DA.

S. No	Annotated Metabolite	Class	Pathway Involved	PLS-DA VIP
1	Trans-4-Coumaric acid	Phenylpropanoid	Ubiquinone and other terpenoid-quinone biosynthesis	1.8064
2	Alpha-tocopherol	Prenol lipid	Ubiquinone and other terpenoid-quinone biosynthesis	1.7903
3	4-Acetyl-2-phenyl-1-pyrolline	Pyrrole	NA	1.7889
4	Farnesol	Isoprenoid	Terpenoid backbone biosynthesis	1.7077
5	Squalene	Phytosterol	Steroid biosynthesis	1.6961
6	Pelargonic acid	Fatty acid	Biosynthesis of unsaturated fatty acids	1.6936
7	GABA	Amino acid	Alanine, aspartate, and glutamate metabolism	1.6808
8	Stigmasterol	Phytosterol	Steroid biosynthesis	1.6785
9	7-Hydroxyflavone	Flavonoid	Flavonoid biosynthesis	1.6394
10	Eicosenoic acid	Fatty acid	Biosynthesis of unsaturated fatty acids	1.635
11	Gluconic acid	Monosaccharide	Pentose phosphate pathway	1.6149
12	Xylitol	Monosaccharide	Pentose and glucuronate interconversions	1.5368
13	Genistein	Flavonoid	Flavonoid biosynthesis	1.4862
14	Campesterol	Phytosterol	Steroid biosynthesis	1.4331
15	Chorismic acid	Carboxylic acid	Ubiquinone and other terpenoid-quinone biosynthesis	1.3917
16	3-Hydroxydecanoic acid	Fatty acid	Biosynthesis of unsaturated fatty acids	1.3803
17	Isovaleric acid	Fatty acid	Biosynthesis of alkaloids	1.3743
18	Glucopyranose	Monosaccharide	Glycolysis	1.3333
19	Beta-Sitosterol	Phytosterol	Steroid biosynthesis	1.2974
20	Isoleucine	Amino acid	Valine, leucine, and isoleucine degradation	1.2964
21	(S)-Malate	Carboxylic acid	Pyruvate metabolism	1.2921
22	L-Leucine	Amino acid	Valine, leucine, and isoleucine degradation	1.2155
23	Spermine	Amino acid	Arginine and proline metabolism	1.2111
24	3,4-Dimethoxycinnamic acid	Carboxylic acid	NA	1.2007
25	L-Pyroglutamic acid	Amino acid	Glutathione metabolism	1.1812
26	1,3-Phenylenediamine	Amine	NA	1.1774
27	Heptadecanoic acid	Fatty acid	Biosynthesis of unsaturated fatty acids	1.1717
28	p-Coumaric acid	Phenylpropanoid	Ubiquinone and other terpenoid-quinone biosynthesis	1.167
29	2-Coumarinate	Phenylpropanoid	Phenylpropanoid biosynthesis	1.1267
30	1-Cyclohexylpyrrolidin-2-one	Pyrrole	NA	1.1239
31	5-Dodecenoic acid	Fatty acid	Fatty acid biosynthesis	1.1156
32	Sinapoyl malate	Phenylpropanoid	Phenylpropanoid biosynthesis	1.1009
33	Sinapoyl aldehyde	Phenylpropanoid	Phenylpropanoid biosynthesis	1.0935
34	2-Linoleoyl-glycerol	Phospholipid	NA	1.0922
35	Gamma-Linolenic acid	Fatty acid	Linoleic acid metabolism	1.0876
36	Caffeic aldehyde	Phenylpropanoid	Phenylpropanoid biosynthesis	1.071
37	p-Coumaraldehyde	Phenylpropanoid	Phenylpropanoid biosynthesis	1.0704
38	Linoleic acid	Fatty acid	Linoleic acid metabolism	1.0614
39	Caffeyl alcohol	Phenylpropanoid	Phenylpropanoid biosynthesis	1.0568
40	Alpha-cyano-4-hydroxycinnamic acid	Carboxylic acid	Ubiquinone and other terpenoid-quinone biosynthesis	1.0485
41	5-Hydroxyconiferaldehyde	Phenylpropanoid	Phenylpropanoid biosynthesis	1.0484
42	L-Valine	Amino acid	Valine, leucine, and isoleucine degradation	1.0215
43	L-Alanine	Amino acid	Alanine, aspartate, and glutamate metabolism	1.0203

**Table 3:** Major pathways discriminating the grain metabolome of Mappillai Samba and CBMAS 14065.

Pathway	Raw *p*	(−log10 (*p*))
Phenylpropanoid biosynthesis	0.000556	3.2552
Ubiquinone and other terpenoid-quinone biosynthesis	0.033338	1.4771
Steroid biosynthesis	0.046969	1.3282

**Table 4:** List of significant nutraceutical and therapeutic metabolites in Mappillai Samba and their potential applications.

S. No	Metabolite	Class	Uses	References
1	β-Sitosterol	Phytosterol	Prevention of cervical cancer; hypocholesterolemic and anti-inflammatory effects	[36]
2	Campesterol	Phytosterol	Antioxidant, hypocholesterolemic, and anti-inflammatory effects	[18,29]
3	Stigmasterol	Phytosterol	Anticancer and cholesterol-lowering ability; reduces the risk of cardiovascular diseases; anti-inflammatory, antioxidant, antiviral, estrogenic, and hypocholesterolemic effects	[15,18]
4	Squalene	Phytosterol	Anticancer, antibacterial, immunostimulant, and cholesterol-lowering ability	[15,18]
5	Trans-4-Coumaric acid	Phenylpropanoid	Antioxidant effect	[37]
6	p-Coumaric acid	Phenylpropanoid	Antioxidant and antimelanogenic effects	[38]
7	Chorismic acid	Carboxylic acid	Key branch-point intermediate for the production of primary and secondary metabolites	[39]
8	7-Hydroxyflavone	Flavonoid	Antioxidant effect	[40]
9	Genistein	Flavonoid	Antitumour effect	[41]
10	Gamma-tocotrienol	Prenol lipid	Potent anticancer agent; lowers cholesterol levels; antiosteoporotic agent	[42,43,44]
11	Alpha-tocopherol	Prenol lipid	Anticancer and antidiabetic effects; anti-infertility, antioxidant, and cardioprotective effects	[18]
12	Spermine	Amino acid	ROS scavenging; protection from stress	[45]
13	Putrescine	Amino acid	Antioxidant effect	[46]

## Data Availability

Data are contained within this article and the Supplementary Information.

## Supplementary Materials

The following are available online at https://www.mdpi.com/article/10.3390/plants11040543/s1, Figure S1: Chromatograms of Mappillai Samba and CBMAS 14065S; Figure S2: Heat map analysis of 113 metabolites in Mappillai Samba and CBMAS 14065; Table S1: List of metabolites identified in Mappillai Samba and CBMAS 14065; Table S2: The abundance ratio of 92 metabolites showed more than a 2-fold change between Mappillai Samba and CBMAS 14065.
