# Supplementary material for: A Comparative Metabolomic Analysis Reveals the Nutritional and Therapeutic Potential of Grains of the Traditional Rice Variety Mappillai Samba
Source: Plants (Basel). 2022 Feb 18;11(4):543. doi: 10.3390/plants11040543 (PMC8876031; doi:10.3390/plants11040543)
Supplement: Supplementary file 1 [file plants-11-00543-s001.zip › plants-1538875-supplementary.pdf]

Supplementary Figure S1. GC-MS/MS chromatogram of methanolic extracts of Mappillai Samba and CBMAS14065 grains.

GC-MS/MS chromatogram of CBMAS 14065 grains

TAMILNADU AGRICULTURAL UNIVERSITY - AGRICULTURAL MICROBIOLOGY

INSTRUMENT : PERKIN ELMER CLARUS SQ8C
INJECTION : 1 MICRO LITER
SAMPLE ID : 65-1 0707

COLOUMN : DB-5 MS CAPILARY STANDARD NON POLARCOLOUMN
DIMENSION : 30Mts, ID: 0.25 mm, FILM: 0.25 IM CARRIER GAS: He

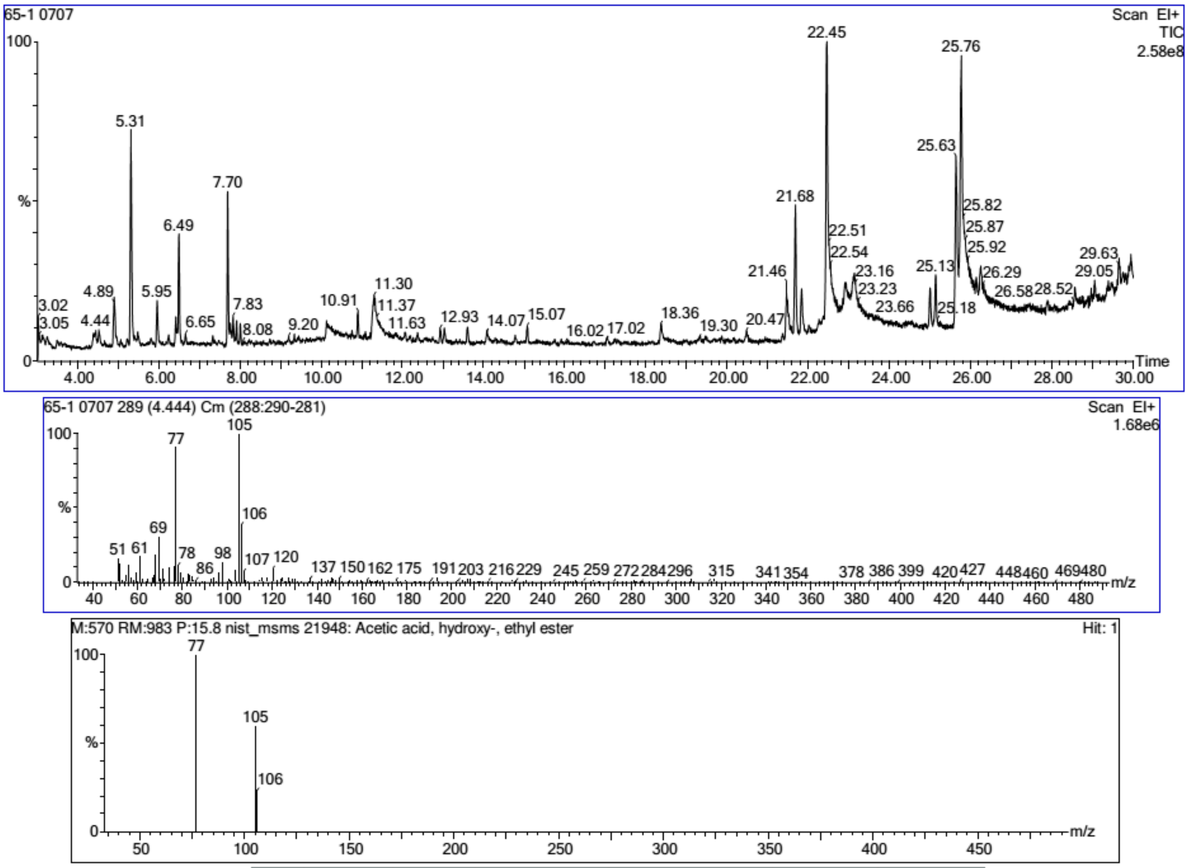

#	RT	Scan	Height	Area	Area %	Norm %
1	4.444	289	14,129,727	751,556.6	0.384	2.81

Pk #	RT	Hit	Compound Name	Match	R.Match	Prob.	CAS	Library
1	4.444	1	Acetic acid, hydroxy-, ethyl ester	570	983	15.8	623-50-7	nist_msms
		2	Benzoic acid 3-methyl-4-(1,3,3,3-tetrafluoro-2-methoxycarbonyl-propenylsulfanyl)-phenyl ester	568	616	14.6		mainlib
		3	3-Fluoro-3-(2-oxo-2-phenyl-ethylsulfanyl)-2-trifluoromethyl-acrylic acid methyl ester	541	601	4.3	329735-34-4	mainlib
		4	Thiophene-2-ol, benzoate	530	844	2.9	16693-98-4	mainlib
		5	N-[5-(Trifluoromethyl)-1,3,4-thiadiazol-2-yl]benzamide	530	692	2.9	54047-24-4	mainlib
		6	2-Hydroxy-2-methyl-succinic acid, bis-(2-oxo-2-phenyl-ethyl ester	515	564	1.8		mainlib
		7	E-2-Hexenyl benzoate	512	606	1.6	76841-70-8	replib
		8	N-[5-(1-Cyano-2-furan-2-yl-vinyl)-[1,3,4]thiadiazol-2-yl]-benzamide	512	587	1.6		mainlib
		9	Propanal, 2-(benzoyloxy)-, (R)-	511	680	1.5	78871-04-2	mainlib
		10	Acetic acid, hydroxy-, ethyl ester	509	922	15.8	623-50-7	nist_msms

GC-MS/MS chromatogram of Mappillai Samba grains

TAMILNADU AGRICULTURAL UNIVERSITY - AGRICULTURAL MICROBIOLOGY

INSTRUMENT : PERKIN ELMER CLARUS 500C
INJECTION : 1 MICRO LITER
SAMPLE ID : MS1 0707

COLOUMN : DB-5 MS CAPILARY STANDARD NON POLARCOLOUMN
DIMENSION : 30Mts, ID: 0.25 mm, FILM: 0.25 µM CARRIER GAS: He

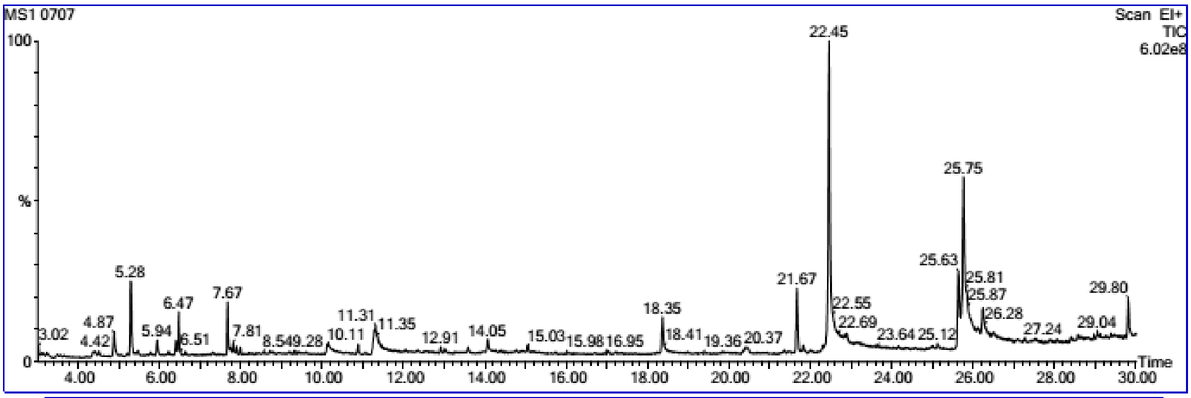

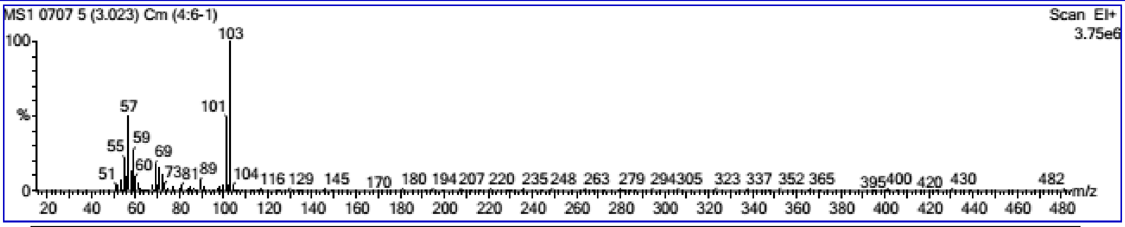

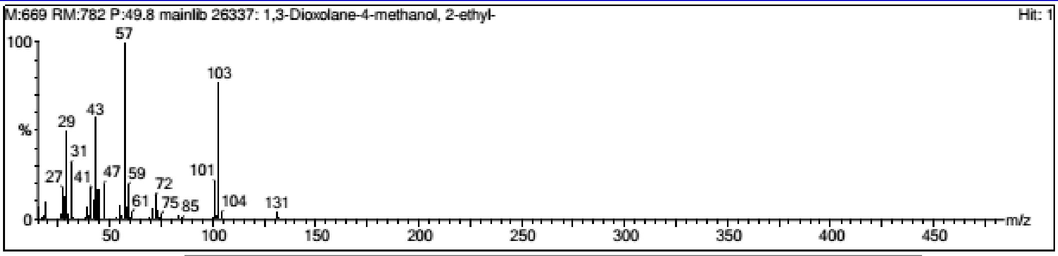

#	RT	Scan	Height	Area	Area %	Norm %
1	3.023	5	27,461,184	837,315.9	0.387	1.82

Pk #	RT	Hit	Compound Name	Match	R.Match	Prob.	CAS	Library
1	3.023	1	1,3-Dioxolane-4-methanol, 2-ethyl-	669	782	49.8	53951-44-3	mainlib
		2	Butane, 1,1-diethoxy-	622	715	10.4	3658-95-5	replib
		3	Methyl 3-hydroxytetradecanoate	606	680	6.0	55682-83-2	replib
		4	Methyl 3-hydroxytetradecanoate	602	679	6.0	55682-83-2	replib
		5	Decanoic acid, 3-hydroxy-, methyl ester	599	625	4.6	56618-58-7	mainlib
		6	Decanoic acid, 3-hydroxy-, methyl ester	596	645	4.6	56618-58-7	replib
		7	Octadecanoic acid, 3-hydroxy-, methyl ester	593	679	3.6	2420-36-2	replib
		8	Hexadecanoic acid, 3-hydroxy-, methyl ester	588	661	2.9	51883-36-4	replib
		9	2,3,4,4-Tetramethyl-pentane-1,3-diol	587	650	2.8		mainlib
		10	Propane, 1,1-diethoxy-2-methyl-	585	641	2.6	1741-41-9	mainlib

Supplementary Figure S2. Heat map of 113 metabolites of Mappillai Samba and CBMAS 14065 grains. First column represents CBMAS 14065 and second column represents Mappillai samba. Red colour shows up-regulation and blue colur indicates down regulation.

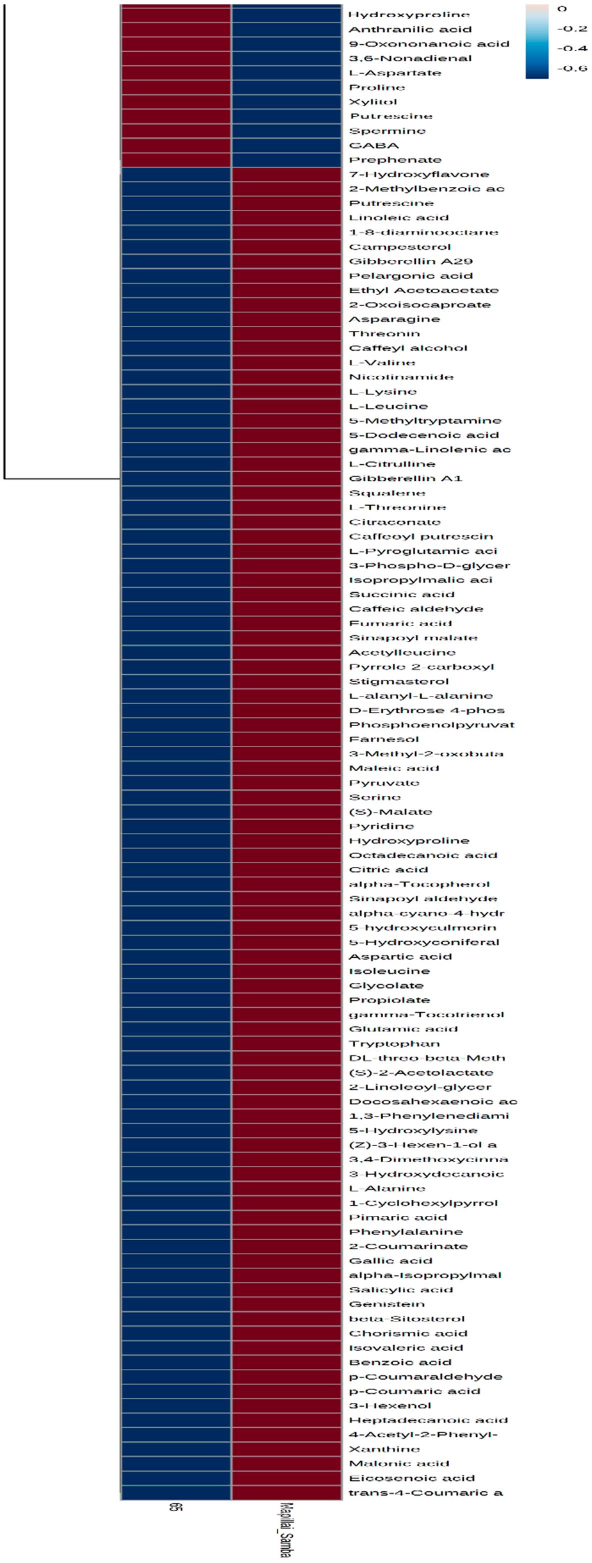

Supplementary Table S1. Details on the metabolites detected in the grains of Mappillai Samba and CBMAS 14065 along with their retention time, peak area, metabolite class and metabolic pathway involved.

S. No	RT (min)	Peak m/z	Annotated metabolites	Class	Pathway involved
1.	27.52	90.09	L-Alanine	Amino acids	Alanine, aspartate and glutamate metabolism
2.	19.77	102.00	GABA	Amino acids	Alanine, aspartate and glutamate metabolism
3.	26.28	132.11	Asparagine	Amino acids	Alanine, aspartate and glutamate metabolism
4.	27.46	191.09	Citric acid	Carboxylic Acids	Alanine, aspartate and glutamate metabolism
5.	27.11	117.02	Succinic acid	Carboxylic Acids	Alanine, aspartate and glutamate metabolism
6.	3.73	138.21	3,6-Nonadienal	Fatty acids	alpha-Linolenic acid metabolism
7.	20.32	171.10	9-Oxononanoic acid	Fatty acids	alpha-Linolenic acid metabolism
8.	21.70	142.20	(Z)-3-Hexen-1-ol acetate	Fatty acids	alpha-Linolenic acid metabolism
9.	29.27	100.09	3-Hexenol	Fatty acids	alpha-Linolenic acid metabolism
10.	21.67	169.01	Gallic acid	Carboxylic Acids	Aminobenzoate degradation
11.	23.29	249.12	Caffeoylputrescin	Amines	Arginine and proline metabolism
12.	23.23	116.06	Proline	Amino acids	Arginine and proline metabolism
13.	6.06	131.18	Putrescine	Amino acids	Arginine and proline metabolism
14.	8.82	203.22	Spermine	Amino acids	Arginine and proline metabolism
15.	22.20	132.00	Hydroxyproline	Amino acids	Arginine and proline metabolism
16.	22.45	68.05	Pyrrole 2-carboxylic acid	Azoles	Arginine and proline metabolism
17.	7.03	265.15	Feruloylputrescine	Carboxylic acids	Arginine and proline metabolism
18.	12.14	133.03	Aspartic acid	Amino acids	Arginine biosynthesis
19.	22.87	148.06	Glutamic acid	Amino acids	Arginine biosynthesis
20.	12.60	159.08	L-Citrulline	Amino acids	Arginine biosynthesis
21.	15.44	115.03	Fumaric acid	Carboxylic acids	Arginine biosynthesis
22.	11.28	68.99	Propiolate	Carboxylic Acids	beta-Alanine metabolism
23.	3.45	103.08	Isovaleric acid	Fatty Acids	Biosynthesis of alkaloids
24.	29.46	74.99	Glycolate	Carboxylic Acids	Biosynthesis of secondary metabolites
25.	29.91	309.40	Eicosenoic acid	Fatty acid	Biosynthesis of unsaturated fatty acids
26.	22.45	157.20	Pelargonic acid	Fatty Acids	Biosynthesis of unsaturated fatty acids

S. No	RT (min)	Peak m/z	Annotated metabolites	Class	Pathway involved
27.	26.52	187.21	3-Hydroxydecanoic acid	Fatty Acids	Biosynthesis of unsaturated fatty acids
28.	21.49	269.30	Heptadecanoic acid	Fatty Acids	Biosynthesis of unsaturated fatty acids
29.	29.92	327.23	Docosahexaenoic acid	Fatty acids	Biosynthesis of unsaturated fatty acids
30.	23.55	304.22	Eicosanoic acid	Fatty acids	Biosynthesis of unsaturated fatty acids
31.	28.78	301.22	Pimaric acid	Diterpenoids	Diterpenoid biosynthesis
32.	4.71	347.57	Gibberellin A1	Diterpenoids	Diterpenoid biosynthesis
33.	3.28	347.08	Gibberellin A29	Diterpenoids	Diterpenoid biosynthesis
34.	6.38	104.01	Malonic acid	Carboxylic Acids	Fatty acid biosynthesis
35.	24.16	197.20	5-Dodecenoic acid	Fatty Acids	Fatty acid biosynthesis
36.	22.77	144.11	Octadecanoic acid	Fatty acids	Fatty acid biosynthesis
37.	24.59	237.05	7-Hydroxyflavone	Flavonoid	Flavonoid biosynthesis
38.	27.22	269.04	Genistein	Flavonoid	Flavonoid biosynthesis
39.	29.55	179.01	Raffinose	Polysaccharides	Galactose metabolism
40.	7.54	128.03	L-Pyroglutamic acid	Amino acids	Glutathione metabolism
41.	23.24	105.04	Serine	Amino acids	Glycine, serine and threonine metabolism
42.	18.45	118.10	Glucopyranose	Monosaccharides	Glycolysis
43.	22.80	184.99	3-Phospho-D-glycerate	Organic phosphate	Glycolysis
44.	25.75	277.22	gamma-Linolenic acid	Fatty acid	Linoleic acid metabolism
45.	21.99	278.22	alpha-Linolenic acid	Fatty Acids	Linoleic acid metabolism
46.	22.49	279.23	Linoleic acid	Fatty Acids	Linoleic acid metabolism
47.	22.49	145.10	L-Lysine	Amino acids	Lysine biosynthesis
48.	12.34	159.07	L-alanyl-L-alanine	Amino acids	Lysine biosynthesis
49.	13.56	134.10	L-Aspartate	Amino acids	Lysine biosynthesis
50.	13.14	163.10	5-Hydroxylysine	Amino acids	Lysine degradation
51.	21.69	109.00	1,3-Phenylenediamine	Amines	NA
52.	22.80	145.17	1-8-diaminooctane	Amines	NA
53.	12.14	146.04	DL-threo-beta-Methylaspartic acid	Amino acids	NA
54.	22.45	172.10	Acetylleucine	Amino acids	NA
55.	21.51	84.02	1H-1,2,3-triazole-5-OH	Azoles	NA

S. No	RT (min)	Peak m/z	Annotated metabolites	Class	Pathway involved
56.	26.99	207.07	3,4-Dimethoxycinnamic acid	Carboxylic Acids	NA
57.	24.55	279.30	2-Linoleoyl-glycerol	Phospholipids	NA
58.	25.73	80.05	Pyridine	Pyridines	NA
59.	10.12	187.09	4-Acetyl-2-Phenyl-1-Pyrolline	Pyrroles	NA
60.	17.05	168.14	1-Cyclohexylpyrrolidin-2-one	Pyrroles	NA
61.	25.75	123.05	Nicotinamide	Amide	Nicotinate and nicotinamide metabolism
62.	10.67	152.06	Xylitol	Monosaccharides	Pentose and glucuronateinterconversions
63.	29.47	195.05	Gluconic acid	Monosaccharides	Pentose phosphate pathway
64.	6.40	139.03	Salicylic acid	Carboxylic Acids	Phenylalanine metabolism
65.	6.77	105.08	Benzoic acid	Carboxylic acids	Phenylalanine metabolism
66.	25.66	166.08	Phenylalanine	Amino acids	Phenylalanine, tyrosine and tryptophan biosynthesis
67.	22.88	173.04	(-)Shikimic acid	Carboxylic Acids	Phenylalanine, tyrosine and tryptophan biosynthesis
68.	24.63	169.00	Phosphoenolpyruvate	Carboxylic acids	Phenylalanine, tyrosine and tryptophan biosynthesis
69.	16.89	226.05	Prephenate	Carboxylic acids	Phenylalanine, tyrosine and tryptophan biosynthesis
70.	16.53	96.97	D-Erythrose 4-phosphate	Sugar Phosphates	Phenylalanine, tyrosine and tryptophan biosynthesis
71.	17.55	147.00	2-Coumarinate	Phenylpropanoids	Phenylpropanoid biosynthesis
72.	20.24	339.31	Sinapoyl malate	Phenylpropanoids	Phenylpropanoid biosynthesis
73.	20.02	209.08	Sinapoyl aldehyde	Phenylpropanoids	Phenylpropanoid biosynthesis
74.	26.14	164.08	Caffeic aldehyde	Phenylpropanoids	Phenylpropanoid biosynthesis
75.	22.56	149.05	p-Coumaraldehyde	Phenylpropanoids	Phenylpropanoid biosynthesis
76.	26.61	165.05	Caffeyl alcohol	Phenylpropanoids	Phenylpropanoid biosynthesis
77.	26.27	194.11	5-Hydroxyconiferaldehyde	Phenylpropanoids	Phenylpropanoid biosynthesis
78.	21.22	237.18	5-hydroxyculmorin	Phenylpropanoids	Phenylpropanoid biosynthesis
79.	22.52	153.09	Xanthine	Alkaloids	Purine metabolism
80.	11.26	133.01	(S)-Malate	Carboxylic Acids	Pyruvate metabolism
81.	21.15	411.39	Squalene	Phytosterols	Steroid biosynthesis
82.	27.41	394.50	Stigmasterol	Phytosterols	Steroid biosynthesis
83.	28.63	400.00	Campesterol	Phytosterols	Steroid biosynthesis
84.	28.11	396.50	β-Sitosterol	Phytosterols	Steroid biosynthesis

S. No	RT (min)	Peak m/z	Annotated metabolites	Class	Pathway involved
85.	26.50	223.20	Farnesol	Isoprenoids	Terpenoid backbone biosynthesis
86.	13.05	175.12	5-Methyltryptamine	Amines	Tryptophan metabolism
87.	25.95	204.08	Tryptophan	Amino acids	Tryptophan metabolism
88.	5.68	87.00	Pyruvate	Carboxylic Acids	Tryptophan metabolism
89.	22.65	139.26	Anthranilic acid	Carboxylic Acids	Tryptophan metabolism
90.	4.86	158.06	Indole-3-acetaldehyde	Indoles	Tryptophan metabolism
91.	25.66	198.08	3,4-Dihydroxy-L-phenylalanine	Amino acids	Tyrosine metabolism
92.	4.55	115.00	Maleic acid	Carboxylic Acids	Tyrosine metabolism
93.	20.69	130.06	Ethyl Acetoacetate	Carboxylic acids	Tyrosine metabolism
94.	18.95	163.06	L-Tyrosine	Amino acids	Ubiquinone and other terpenoid-quinone biosynthesis
95.	26.76	212.03	alpha-cyano-4-hydroxycinnamic acid	Carboxylic Acids	Ubiquinone and other terpenoid-quinone biosynthesis
96.	22.48	227.06	Chorismic acid	Carboxylic acid	Ubiquinone and other terpenoid-quinone biosynthesis
97.	14.37	163.04	trans-4-Coumaric acid	Phenylpropanoids	Ubiquinone and other terpenoid-quinone biosynthesis
98.	12.07	165.05	p-Coumaric acid	Phenylpropanoids	Ubiquinone and other terpenoid-quinone biosynthesis
99.	21.22	430.39	alpha-Tocopherol	Prenol lipids	Ubiquinone and other terpenoid-quinone biosynthesis
100.	28.15	416.35	gamma-Tocotrienol	Prenol lipids	Ubiquinone and other terpenoid-quinone biosynthesis
101.	6.24	120.09	Threonin	Amino acids	Valine, leucine and isoleucine biosynthesis
102.	18.06	103.06	L-Threonine	Amino acids	Valine, leucine and isoleucine biosynthesis
103.	14.09	129.09	Citraconate	Carboxylic Acids	Valine, leucine and isoleucine biosynthesis
104.	21.36	175.05	Isopropylmalic acid	Carboxylic Acids	Valine, leucine and isoleucine biosynthesis
105.	14.97	177.08	alpha-Isopropylmalate	Carboxylic acids	Valine, leucine and isoleucine biosynthesis
106.	10.68	133.07	(S)-2-Acetolactate	Carboxylic acids	Valine, leucine and isoleucine biosynthesis
107.	16.62	89.03	2-Oxoisocaproate	Carboxylic acids	Valine, leucine and isoleucine biosynthesis
108.	18.69	147.07	Citramalate	Carboxylic acids	Valine, leucine and isoleucine biosynthesis
109.	26.11	131.20	Isoleucine	Amino acids	Valine, leucine and isoleucine degradation
110.	7.69	172.10	L-Leucine	Amino acids	Valine, leucine and isoleucine degradation
111.	27.57	134.96	L-Valine	Amino acids	Valine, leucine and isoleucine degradation
112.	9.44	150.07	2-Methylbenzoic acid	Carboxylic acids	Valine, leucine and isoleucine degradation
113.	5.35	115.07	3-Methyl-2-oxobutanoic acid	Carboxylic Acids	Valine, leucine and isoleucine degradation

Supplementary Table S2. Abundance ratio of 92 metabolites showing > 2 fold difference between the grains of Mappillai Samba and CBMAS 14065.

S. No	Metabolites	Fold Change (FC)	log2 (FC)
1.	trans-4-Coumaric acid	477.33	8.8988
2.	L-Leucine	333.69	8.3824
3.	Chorismic acid	270.43	8.0791
4.	4-Acetyl-2-Phenyl-1-Pyrolline	215.52	7.7517
5.	7-Hydroxyflavone	155.27	7.2786
6.	5-hydroxyculmorin	134.59	7.0724
7.	gamma-Tocotrienol	133.4	7.0597
8.	p-Coumaric acid	126.84	6.9869
9.	Eicosenoic acid	95.37	6.5755
10.	3-Hydroxydecanoic acid	93.104	6.5408
11.	Campesterol	92.06	6.5245
12.	alpha-Tocopherol	76.196	6.2516
13.	Pelargonic acid	74.62	6.2215
14.	Farnesol	63.811	5.9957
15.	Isoleucine	61.502	5.9426
16.	L-Tyrosine	40.028	5.3229
17.	1,3-Phenylenediamine	38.41	5.2634
18.	1-Cyclohexylpyrrolidin-2-one	35.318	5.1423
19.	(S)-Malate	34.926	5.1262
20.	Stigmasterol	33.086	5.0481
21.	Genistein	32.536	5.024
22.	Squalene	29.328	4.8742
23.	5-Dodecenoic acid	29.255	4.8706
24.	Heptadecanoic acid	27.424	4.7774
25.	5-Hydroxyconiferaldehyde	21.339	4.4154
26.	Phenylalanine	21.317	4.4139
27.	L-Pyroglutamic acid	20.295	4.3431
28.	Caffeyl alcohol	19.159	4.2599
29.	Succinic acid	18.15	4.1819
30.	Sinapoyl aldehyde	17.191	4.1036
31.	3,4-Dimethoxycinnamic acid	16.843	4.074
32.	Threonin	16.555	4.0492
33.	p-Coumaraldehyde	16.385	4.0343
34.	2-Coumarinate	15.486	3.9529
35.	Pyrrole 2-carboxylic acid	14.969	3.9039
36.	Asparagine	14.467	3.8547
37.	2-Linoleoyl-glycerol	13.903	3.7974
38.	Glutamic acid	13.891	3.7961
39.	Pyridine	12.574	3.6524
40.	Sinapoyl malate	11.997	3.5846
41.	Propiolate	11.841	3.5657

42.	L-Valine	11.793	3.5599
43.	Serine	10.186	3.3485
44.	5-Methyltryptamine	9.5614	3.2572
45.	Nicotinamide	9.5006	3.248
46.	1H-1,2,3-triazole-5-OH	8.7529	3.1298
47.	β-Sitosterol	8.5232	3.0914
48.	Aspartic acid	8.4485	3.0787
49.	L-Threonine	8.3906	3.0688
50.	1-8-diaminooctane	8.2666	3.0473
51.	Linoleic acid	7.8498	2.9727
52.	alpha-cyano-4-hydroxycinnamic acid	7.6177	2.9294
53.	Glycolate	7.3426	2.8763
54.	gamma-Linolenic acid	7.1343	2.8348
55.	Putrescine	6.7482	2.7545
56.	L-alanyl-L-alanine	6.3864	2.675
57.	3-Hexenol	6.3674	2.6707
58.	L-Alanine	6.142	2.6187
59.	Caffeoylputrescin	5.9238	2.5665
60.	3-Methyl-2-oxobutanoic acid	5.7424	2.5216
61.	Maleic acid	5.365	2.4236
62.	Xanthine	5.3233	2.4123
63.	Tryptophan	5.3069	2.4079
64.	Proline	5.0462	2.3352
65.	(S)-2-Acetolactate	4.9057	2.2945
66.	Gallic acid	4.8018	2.2636
67.	Isopropylmalic acid	4.7703	2.2541
68.	Pimaric acid	4.6171	2.207
69.	Caffeic aldehyde	4.5421	2.1834
70.	Malonic acid	3.5519	1.8286
71.	2-Methylbenzoic acid	3.1749	1.6667
72.	5-Hydroxylysine	3.1719	1.6654
73.	Fumaric acid	3.1484	1.6546
74.	9-Oxononanoic acid	3.1366	1.6492
75.	Eicosanoic acid	2.8766	1.5243
76.	Acetylleucine	2.8309	1.5013
77.	Isovaleric acid	2.8098	1.4905
78.	Benzoic acid	2.7639	1.4667
79.	2-Oxoisocaproate	2.6787	1.4216
80.	Anthranilic acid	2.4538	1.295
81.	Citraconate	2.4493	1.2923
82.	L-Citrulline	2.3453	1.2298
83.	Salicylic acid	2.3235	1.2163
84.	Docosahexaenoic acid	2.2166	1.1484
85.	Raffinose	0.40299	-1.3112
86.	L-Aspartate	0.36263	-1.4634
87.	Spermine	0.27234	-1.8765

88.	Hydroxyproline	0.069276	-3.8515
89.	GABA	0.062121	-4.0088
90.	Xylitol	0.029718	-5.0725
91.	Gluconic acid	0.025259	-5.307
92.	Glucopyranose	0.020689	-5.595
